# Weight Self-Regulation Process in Adolescence: The Relationship between Control Weight Attitudes, Behaviors, and Body Weight Status

**DOI:** 10.3389/fnut.2015.00014

**Published:** 2015-05-08

**Authors:** Jordi Pich, Maria del Mar Bibiloni, Antoni Pons, Josep A. Tur

**Affiliations:** ^1^Research Group on Community Nutrition and Oxidative Stress, CIBEROBN (Physiopathology of Obesity and Nutrition CB12/03/30038), University of the Balearic Islands, Palma de Mallorca, Spain

**Keywords:** adolescents, body image, overweight, obesity, weight self-control, dieting, exercising

## Abstract

Adolescents’ self-control weight behaviors were assessed (*N* = 1961; 12–17 years old; 2007–2008) in the Balearic Islands, Spain. The study analyzed the relationships between body weight status, body image, and self-weight concern, and actual attempts to lose weight by restrained eating and/or increased exercising. In terms of regulatory focus theory (RFT), we considered that efforts to lose or to maintain weight (successful or failed) would be motivated either by a “promotion focus” (to show an attractive body), or a “prevention focus” (to avoid social rejection of fatness), or both. Results showed that 41% of overweight boys and 25% of obese boys stated that they had never made any attempt to lose weight, and 13 and 4% in females. Around half of overweight boys and around a quarter of obese boys stated that they were “Not at all” concerned about weight gain, and girls’ percentages decreased to 13 and 11%, respectively. By contrast, 57% of normal weight girls monitored their weight and stated that they had tried to become slim at least once. Weight self-regulation in females attempted to combine diet and exercise, while boys relied almost exclusively on exercise. Apparent lack of consciousness of body weight status among overweight boys, and more important, subsequent absence of behaviors to reduce their weight clearly challenges efforts to prevent obesity. We argue that several causes may be involved in this outcome, including unconscious, emotional (self-defense), and cognitive (dissonance) mechanisms driven by perceived social stigmatization of obesity. The active participation of social values of male and female body image (strong vs. pretty), and the existence of social habituation to overweight are suggested. A better knowledge of psychosocial mechanisms underlying adolescent weight self-control may improve obesity epidemics.

## Introduction

Results from US National Health and Nutrition Examination Surveys (NHANES) pointed out that the obesity prevalence remained stable over past 10 years (1). However, several cross-surveys in Western countries showed that around 30% of adolescents boys and 25% of girls are still overweighed (2, 3), mainly due to high-calorie food intake and sedentary lifestyle.

The increased prevalence in adolescent overweight contrasts paradoxically with the prevailing social appreciation of thinness (4–6), which is more pronounced among females (7, 8). The flip side of that appreciation is society’s rejection of obesity, whether expressed openly or through implicit attitudes (9).

Longitudinal surveys and psychiatric studies have long warned that adolescents’ weight self-regulation practices promoted by thinness goals sometimes fuel unhealthy habits such as smoking, alcohol and drug use, purges, or vomits, or even result in anorexia and bulimia disorders (10).

Comprehensible alarm resulting from these potential hazards has tended to obscure the positive role that body weight self-regulation processes, motivated by concern with body image, may represent by promoting a more balanced food intake and regular physical activity during adolescence. In our view, the relationship between ideal body image and weight self-regulation process can be connected through Higgins’ (11, 12) regulatory focus theory (RFT). Essentially, RFT describes the processes by which individuals try to self-regulate or adjust their attitudes and behaviors to achieve a positive goal by means of two differentiated strategies: promotion focus goals (“making good things happen”) or prevention focus goals (“avoiding bad things happen”). Some studies on adults have explored the relationship between individuals’ dominant focus and several dimensions of eating behavior, such as fruit consumption (13), restrained eating (14), emotional, external and restrained eating (15), or food choice motives (16).

In this theoretical framework, we consider that adolescents’ weight self-regulation efforts either by losing excessive weight, or by maintaining it in reasonable magnitudes, would involve a motivational “promotion focus” (i.e., the desire to show an attractive personal body image in alignment with present beauty standards) as well as a “prevention focus” (i.e., to avoid social rejection linked to deviance of body standards).

However, as any human motivated behavior, weight-control may turn into a serious mental illness (17). Anorexia would represent the most dramatic case of healthy weight self-regulation failure. On the other side, adolescents’ hypothetical involvement in a promotion focus, a prevention one, or both can contribute to initiating positive attempts to eat a more balanced diet and/or practice regular physical activity. In this direction, after remarking that during adolescence, body attractiveness tends to be a stronger motive than health when adopting healthy habits; Nowak (18) observed that boys and girls who attempted weight loss reduced consumption of sweet foods and snacks, while concurrently increasing consumption of healthy foods, such as fruit and yogurt. More recently, another study has also recorded that most obese and overweight boys and girls who manifested their desire to slim reported a congruently healthy lower consumption of several high-calorie food groups (19).

Until now, many cross-sectional surveys have assessed the relationship between losing weight behaviors and body image attitudes. These studies have pointed out the common inclination of the obese and overweight population to underestimate their weight (20–23). On the same lines, some longitudinal studies also have pointed out a propensity to judge their body image as not being excessively overweight, which seems to be consistent with a general decline in students’ dissatisfaction with their body-image (24). In particular, Anglo-American countries have registered a significant increase in the percentage of overweight subjects, in both adults (25, 26) and adolescents (27, 28), who defined their weight as “normal.” In previous studies, roughly half of the overweight boys and a quarter of obese ones also claimed to be normal weight, a belief that was not shared and dropped sharply among girls (20, 21).

With reference to measured body fat, half of the girls with above-average values also considered themselves to be normal weight (29), and 4% even defined themselves as underweight. Contrarily, 33% of normal-weight girls overestimated their weight (30).

In agreement with these unexpected findings within a context of obesity rejection, one longitudinal study found a dip in interest among teenagers in controlling their weight (31). Moreover, in a multi-ethnic sample of British adolescents, 65% of overweight boys and 36% of obese boys stated that they have never tried to lose weight, and these proportions fell to 41 and 23%, respectively, among girls (21). By contrast, the ideal of being slim has been shown to spur girls with normal weight to perceive themselves as “fat” (20, 23), and most of them admit to have dieted on some occasions (21).

Incongruences between self-regulatory theoretical predictions and empirical findings demand a better understanding of self-weight goals among adolescents. The aim of this study was to assess the relationship between body mass status, body image perception, self-weight concern, and the personal motivation to actually engage in healthy weight control behaviors through cutting down food intake and/or regular exercise in a representative sample of adolescents living in a Mediterranean area. Body image satisfaction according to weight status, age, and gender was also assessed.

## Materials and Methods

### Study design and population

The study is part of a broader population-based cross-sectional survey carried out on Balearic Islands’ 12- to 17-year-old adolescents between 2007 and 2008. The sample was selected by means of a multiple-step, simple random sampling, taking into account, first, the location, with towns from all over the Balearic Islands being represented (Palma de Mallorca, Calvià, Inca, Manacor, Maó, Eivissa, Llucmajor, Santa Margalida, S’Arenal, Sant Jordi de Ses Salines), and then by random selection of schools within each town. Sample size was stratified by age and gender.

To calculate a representative number of adolescents, a variable BMI was selected with the greatest variance for this age group, based on data from the literature at the time of the study (32). Sampling was determined for the distribution of this variable, with a confidence interval (CI) established at 95% with a ±0.25 error. The total number of subjects (2400) were uniformly distributed within the towns and proportionally distributed by gender and age. Exclusion criteria were: type 2 diabetes, pregnancy, alcohol or drug abuse, and non-directly related nutritional medical conditions.

The sample was oversized to prevent loss of information and was needed to do the fieldwork in complete classrooms. In each school, classrooms were randomly selected from among those of the same grade, or level, and all the adolescents in one classroom were asked to participate in the survey. A letter informing the nature and purpose of the study was sent to parents or legal guardians. After receiving their written consent, the adolescents were then considered for inclusion in the study. All responses to questionnaires were filled in by the adolescents. Once the field study had been completed, the adolescents who did not fulfill the inclusion criteria were excluded. Finally, the sample was adjusted by a weighting factor in order to balance the sample according to the distribution of the Balearic Islands’ population and to guarantee that each of the groups, already defined by the previously mentioned factors (age and gender), were representative. The final number of subjects included in the study, 1961 adolescents (82% participation; 47.9% male), were a representative sample of the Balearic Islands’ adolescent population. Reasons for not taking part were: (a) the subject declined to be interviewed, and (b) the parents did not authorize the interview.

The study was conducted according to the guidelines laid down in the Declaration of Helsinki, and all procedures involving human subjects were approved by the Balearic Islands’ Ethics Committee (Palma de Mallorca, Spain) under number IB-530/05-PI. Written informed consent was obtained from all subjects and also from the next of kin, careers, or guardians on the behalf of the minors involved in the study.

### Body composition

Height was calculated to the nearest millimeter using a mobile anthropometer (Kawe 44444, Kirchner & Wilhelm GmBH Co., KG, Asperg, Germany), with the subject’s head placed in the Frankfurt plane. Body weight was determined to the nearest 100 g using a digital scale (Tefal, sc9210, Groupe SEB, Rumilly, France). The subjects were weighed barefoot, wearing light underwear, as previously described (33). BMI was computed as weight (kg) divided by height (m^2^), and study participants were specifically categorized by age and gender using the BMI cut-offs developed and proposed by the International Obesity Task Force (IOTF) (34) and Cole et al. (35, 36): normal weight: 18.5 ≥ BMI < 24.9; overweight: 25.0 ≥ BMI < 29.9; obesity: BMI ≥ 30.

### Self-reported body weight

The subjects were asked to estimate their current height and weight prior to measurement. Estimates within ±2 kg of real weight were classified as correct, <2 kg under real weight were considered as an underestimate, and >2 kg than real weight were considered as an overestimate.

### Body image perception

**Figure 1:**
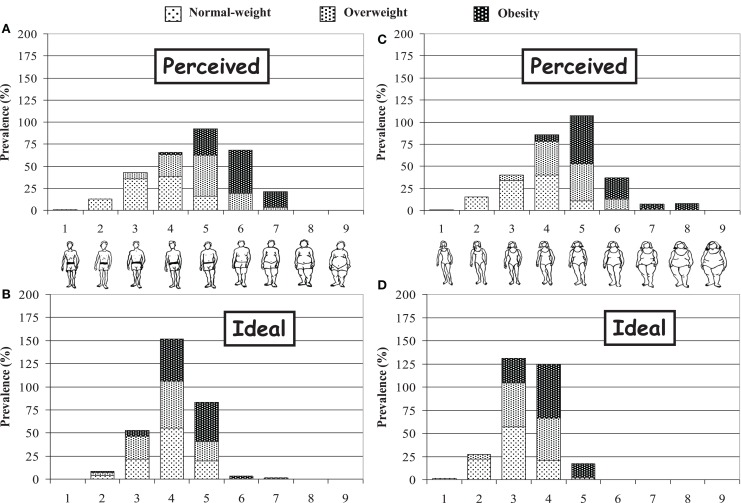
**Perceived and ideal silhouettes chosen by boys (A,B) and girls (C,D)**.

Subjects had to choose the most similar silhouette to their image (“real silhouette”), and the silhouette they would like to have from other similar series (“ideal silhouette”) using Stunkard’s Figure Rating Scale (Figure 1), which includes nine different body silhouettes (37). The difference between the two values was classified as acceptance of body image (ideal = real), dissatisfaction with being overweight (ideal thinner than real), and dissatisfaction with being underweight (ideal weightier than real).

### Attitudes toward self-body weight

The subjects were given multiple choice answers to the question, “Would you say that to gain weight is something that worries you? Not at all – Somewhat – A lot,” followed by the question and multiple choice answers, “I consider myself obese: Yes – No.”

### Behaviors toward body weight self-control

The subjects responded to the question, “Have you ever tried to control your weight? (Yes – Never)” followed by the question, “If you answered yes, have you done so by: dieting (Yes – No); regular sport practice (Yes – No).”

### Statistics

Analyses were performed with the Statistical Package for the Social Sciences, version 21.0 (SPSS, Inc., Chicago, IL, USA). Significant differences in prevalence were calculated by means of χ^2^. The level of significance for acceptance was *P* < 0.05.

## Results

As shown in Table 1, a quarter of the sample showed higher weight than recommended. Around 20% of adolescents were overweight and around 6% were obese, and the incidence in both categories did not differ between genders and ages. Underweight adolescents just represented 0.8% of total sample. Therefore, they were analyzed together with normal weight adolescents.

**Table 1:** **BMI distribution in the sample of adolescents according to the different variables in the study**.

Variables	*n*	BMI (kg/m^2^)			*P*
		Normal weight (18.5 ≥ BMI < 24.9)	Overweight (25.0 ≥ BMI < 29.9)	Obese (BMI ≥ 30)	
Total	1961	74.5	19.6	5.9	
Gender					
Male	939	72.4	20.9	6.7	0.103
Female	1022	76.5	18.4	5.1	
Age (years)					
12–13	495	72.3	21.2	6.5	0.096
14–15	948	73.1	20.5	6.4	
16–17	518	79.1	16.5	4.4	

*Values are %. Significant trends evaluated by χ^2^*.

Figure 1 shows body image self-perceptions. Adolescents tended to attribute silhouettes to themselves with lower signs of body fat than was inferred from the BMI measured. Around 35% of overweight girls ranked themselves as a 4 – normal weight appearance – while almost half of the boys ranked themselves as a 5, a more robust size, but still with no clear signs of being overweight. The same parameters increased by one size among obese adolescents. Moreover, the choice of ideal silhouettes tended to be a larger size in boys, and rose consistently in line with BMI.

Table 2 shows that one-tenth of the subjects did not specify their weight. “Don’t know” answers increased with age and BMI, reaching the 27.8% in the obese group. Weight underestimations increased as BMI increased but decreased with age, and the opposite occurred with overestimation. Girls estimated their weight better than boys, a knowledge that increased with age.

**Table 2:** **Weight estimation, weight gain concern, and body image satisfaction by gender, age, and BMI group**.

Variables^a^	*N*	Weight estimation					Weight gain concern				Body image satisfaction			
		Underestimate	Correct	Overestimate	Don’t know/don’t answer	*P*^b^	Not at all	A little	A lot	*P*^b^	Thinner	Satisfied	Bigger	*P*^b^
Total	1961	27.8	51.9	9.2	11.1		50.4	36.5	13.1		46.8	37.0	16.2	
Gender						<0.001				<0.001	35.4	38.9	25.7	<0.001
Male	939	29.6	48.0	11.7	10.7		65.9	28.0	6.1		57.4	35.2	7.4	
Female	1022	26.1	55.4	6.9	11.5		36.1	44.3	19.6					
Age (years)						0.011				0.071				0.095
12–13	495	32.7	49.6	8.8	8.8		53.6	36.2	10.3		44.4	41.7	14.0	
14–15	948	26.8	52.6	8.6	12.0		47.5	37.9	14.6		48.4	35.4	16.2	
16–17	518	25.1	52.9	10.5	11.5		52.5	34.2	13.2		46.2	35.6	18.3	
BMI (kg/m^2^)						<0.001				<0.001				<0.001
Normal weight	1462	22.1	58.7	10.9	8.3		58.1	31.5	10.4		33.7	44.9	21.4	
Male^c^	680	23.6^NS^	53.7**	14.3***	8.4^NS^		76.5***	19.5***	4.0***		18.1***	46.6^NS^	35.3***	
Female	782	20.8	62.9	8.1	8.2		42.4	41.7	15.9		47.1	43.3	9.6	
Overweight	384	43.6	39.7	5.6	11.1		28.7	50.1	21.1		82.9	16.6	0.6	
Male^c^	196	42.9^NS^	42.3^NS^	7.1^NS^	7.7*		42.2***	48.3^NS^	9.4***		75.7***	23.2**	1.1^NS^	
Female	188	44.4	37.1	3.9	14.6		14.9	52.0	33.1		90.3	9.7	0.0	
Obese	115	51.9	19.4	0.9	27.8		19.8	55.7	24.5		98.1	0.9	0.9	
Male^c^	63	54.2^NS^	16.9^NS^	0.0^NS^	28.8^NS^		23.2^NS^	58.9^NS^	17.9^NS^		96.5^NS^	1.8^NS^	1.8^NS^	
Female	52	49.0	22.4	2.0	26.5		16.0	52.0	32.0		100.0	0.0	0.0	

*^a^Values are expressed as percentages*.

*^b^Significant trends between weight estimation, weight gain concern, and body image satisfaction groups have been evaluated by χ^2^*.

*^c^Significant trends between males and females have been evaluated by χ^2^*.

*P < 0.05; **P < 0.01; ***P < 0.001; NS: not significant.

The difference between the real and ideal silhouette selected by each subject is shown in the body image satisfaction columns, which reveal that nearly half of the teenagers would like to be thinner. However, 25.7% of boys would like to have a larger silhouette.

The percentages of satisfied boys and girls were similar among subjects of normal weight, but girls still wished to be thinner and boys to be larger. Acceptance of being overweight was significantly higher amongst boys.

The Weight gain concern columns in Table 2 clearly reflect girls’ greater concern about gaining weight, which was not affected by age but increased with BMI. Specifically, 47 and 21% of overweight and obese boys, respectively, stated that they were “Not at all” worried about weight concern. These percentages were significantly lower in girls. This unexpected lack of concern about their weight helps to explain the matching lack of any behavior attempt to lose weight in the corresponding column. In effect, 41% of overweight boys and 25% of obese boys stated that they had “Never” attempted to lose weight, and these percentages decreased significantly among females.

Table 3 shows that half of adolescents declared that they had tried to lose weight at least once. This general pattern to lose weight fits for the combination of diet and exercise, but the evolution is quite different when focus is just addressed to exercise, them most of the youngest age group (12-13), and normal weight boys tried more to lose weight with physical activity only.

**Table 3:** **Influence of gender, age, and gender within each BMI group on the intention to lose weight and the start of diet and/or exercise**.

Variables^a^	*N*	Intention	*P*^b^	Do nothing	Diet	Exercise	Diet + Exercise	*P*^b^
Total	1961	48.4		0.6	24.2	38.2	36.9	
Gender			<0.001					<0.001
Male	939	32.9		1.3	11.2	56.8	30.7	
Female	1022	62.7		0.3	30.4	29.3	39.9	
Age			0.004					<0.001
12–13	495	41.8		1.5	18.0	52.5	28.0	
14–15	948	50.3		0.4	26.8	33.3	39.5	
16–17	518	51.2		0.4	24.3	36.1	39.2	
BMI (kg/m^2^)			<0.001					0.001
Normal weight	1462	38.7		0.8	23.9	41.7	33.6	
Male^c^	680	19.2***		1.7	6.7	69.2	22.5***	
Female	782	55.3		0.5	29.0	33.7	36.9	
Overweight	384	74.6		0.0	25.3	37.0	37.7	
Male^c^	196	62.8***		0.0	12.4	48.7	38.9***	
Female	188	86.9		0.0	34.9	28.3	36.8	
Obese	115	88.7		0.0	23.4	20.2	56.4	
Male^c^	63	82.1*		0.0	19.6	37.0	43.5***	
Female	52	96.0		0.0	27.1	4.2	68.8	

*^a^Values are expressed as percentages*.

*^b^Significant trends between intention to lose weight and the start of diet and/or exercise groups have been evaluated by χ^2^*.

*^c^Significant trends between males and females have been evaluated by χ^2^*.

*P < 0.05; ***P < 0.001.

## Discussion

Among the Balearic Islands’ adolescents, the prevalence of overweight is higher and the prevalence of obesity is lower than in the Spanish adolescent population (38). Our study points to important gender differences in weight self-regulation attitudes and behaviors arose in the adolescent period. These findings agree with previous research (39, 40), and are in line with the superior worth given to thinness among girls, a phenomenon usually attributed to different beauty standards in boys and girls (8).

Our data confirm that girls have a more accurate knowledge of their weight than boys (probably by checking it frequently at home); they desire a thinner ideal body image; are much more concerned about weight gain and; as in adults (41), they make more efforts to keep their weight under control. At the studied ages, it was more likely to underestimate than overestimate, similarly to previous findings (42). The obtained results showed that underestimation decreased and overestimation increased with adolescent age. It could be inferred that as much older the adolescent is, as much worried on body weight may be. However, obtained answers on weight gain concern and body image satisfaction did not show it.

According to RFT, the personal motivation to weight self-control may be triggered by the wish to be attractive and/or to avoid to be rejected, being last case more influential, as an individual perceives himself/herself more deviant from social body standard.

However, it is a well-known psychological principle that losing weight is an objective hard to attain, as our adolescents’ figures actually reveal. Clearly, changing comforting habits requires difficult psychological skills, which include an ability to tolerate uncomfortable internal reactions due to hunger or fatigue and a reduction of pleasure, as well as and a behavioral commitment to clearly defined values (43). Therefore, cognitive and emotional expectations about the sacrifices demanded by weight self-regulatory behaviors may dissuade many male and female candidates, while unpleasant experiences from diet and exercise probably lead others to surrender in their first attempts.

This failure may result in frustration and anger (44), a decrease in self-efficacy (45), or even to eat more (46, 47). These psychological outcomes usually arise when people feel unable to control themselves as they wish, or as societal rules dictated (17). In fact, changes in self-regulatory cognitive processes have been demonstrated in the context of adult overeating problems (48, 49).

However, in a social context where the stigma of obesity is a long way of being relaxed (50), the high percentages particularly of overweight males seemingly unconscious of their weight status, unconcerned about weight gain, and consequently unwilling to lose weight, were not. Accordingly, in agreement with similar studies, it can be concluded that most overweight and many obese boys seem to be satisfied with their physical appearance. Therefore, according to the Transtheorical Model (TTM) stages of change (51, 52), this adolescent population should be located in the “pre-contemplation step,” i. e., healthy weight loss practices are not to be expected if subjects do not recognize excess weight, do not consider overweight to be a problem, or that is not a problem serious enough to engage in demanding weight self-regulatory behaviors.

Looking for alternative explanations, one may conjecture that the obese stigmatization actually threatens the self-esteem, particularly of the most weighted individuals, thus setting up subconscious ego self-defense denial mechanisms to protect self-esteem (53–55). High percentage of obese who declared that they did not know their weight would fit this interpretation.

Moreover, social stigma may also pose a cognitive dissonance conflict to overweighed population. Thus, biased body image perception and weight underestimations might be a reflection of a compromise between the social “ought” to body self-image and the perceived body self-image. In other words, self-indulgent weight judgments would be attempts to restore cognitive consonance by claiming “I’m not so fat!” This would explain why half of overweight and obese boys underestimated their weight.

However, our data reveal that most overweight and obese girls do not seem to be affected by the same emotional and cognitive processes. Therefore, beyond the superior worth given to thinness among girls, other psychological parameters are needed to explain the high percentage of boys not concerned about weight gain, or even willing to gain it (65.9 and 25% respectively). Because in male population, masculinity is frequently correlated with a big body, many boys may have considered that to look “manly,” one should better be “well built” than “slim.” This aim at looking “strong” parallels the girls’ concern at looking “pretty.” Thus, in the case of males, being overweight is not always associated to a negative social value, but rather to a positive one if associated to strength. Accordingly, to be a very thin boy may trigger more negative social consequences that to be moderately overweight (luckily, the percentage of underweight boys in our sample was irrelevant). Indeed, a silhouette that represents a slightly overweight boy could be interpreted as a silhouette representing a muscular boy (i.e., a “well-built” boy). This could also explain the discrepancy between the ideal silhouettes chosen by boys and girls.

In conclusion, “the lesser of two evils principle” is somewhat pertinent in this context: when confronted to the choice “being a bit overweight” vs. being a “bit too thin,” perhaps, most boys would choose the first option.

Complementarily, our results could indicate the emergence of a process of social habituation to people exhibiting signs of overweight, a phenomenon comparable to the familiarity with shaved heads, which made it acceptable to be bald. In this direction, Rand and Resnick (56) not only asked teenagers to choose a real and ideal silhouette from a series, but also to check those that were socially acceptable. This study reported that 85% of overweight teenagers and 54% of obese ones considered that their current shape was within the socially acceptable margins of body size, regardless of whether or not they would like to have a slimmer figure. These results are compatible with the social habituation process hypothesis, which deserves further research including estimations of the “regular” or “average” silhouette. Moreover, new researches on body image attitudes demand a better methodology, thus substituting the classical but rather imprecise drawings we employed (e.g., silhouette that represents a slightly overweight boy could be interpreted as a silhouette representing a muscular boy). Instead, the employ of distorted pictures of the subjects (57) asking them to adjust on a computer screen should be encouraged.

Beyond the influence of body image attitudes on weight self-control, it has recently been suggested that personality factors, such as impulsivity and reward/punishment sensitivity, may also play a role in the observed gender motivational and behavioral differences in weight self-monitoring and control (58). The perfectionism personality trait has also previously been related to weight self-regulation, with adult women, who are highly perfectionistic, being more likely to see themselves as overweight and to be more dissatisfied with their bodies (59).

Nowadays, when public efforts to prevent overweight are multiplying (60–62), and many specific school-based interventions have been addressed to cope with the problem (63, 64), a deeper comprehension of factors involved in adolescent weight self-control may contribute to increase their efficiency. Accordingly, our results suggest that to stimulate the use of tailored messages regarding different boys and girls, body image attitudes could be beneficial to obesity epidemics.

Moreover, messages must face the fact that many overweight and obese boys do not actually feel the necessity to lose weight, rather to attribute their physical status to laziness, lack of will-power, or other common negative social stereotypes of overweight people (50).So, in the field of obesity prevention, researches should bear in mind Obelix’s [a character from a well-known French comic book, (65)] famous denial, “I’m not fat. My chest just slipped a bit!,” and adapt their messages suitably.

Another practical finding of our study is the important gender difference we observe in behaviors to achieve a healthy weight. In fact, girls typically used diet and exercise to lose weight, and boys used only exercise. The different competing body image motivations between boys and girls (e.g., to “look like strong” vs. to “look like pretty”) probably boosts the males’ link between sport and muscles and the females’ one between diet and slenderness, thus explaining the girls’ increased confidence in diet and the boys in exercise. Moreover, boys are perhaps more reluctant to go hungry, and girls have a greater fear of having their figures criticized. Therefore, interventions to prevent obesity should suitably accommodate and highlight the benefits of diet among boys, and physical activity among girls.

Afterward, interventions should focus on the advantages of maintaining a reasonable weight by healthy eating and exercise habits by means of presenting well described, attractive strategies to achieve that goal.

## Author Contributions

JT, MB, AP, and JP conceived, designed, devised and supervised the study. MB, JP, and JT collected and supervised the samples. MB, AP, and JT analyzed the data and JP, MB, and JT wrote the manuscript. AP and JT obtained funding. All authors read and approved the final manuscript. The study sponsor had no role in study design.

## Conflict of Interest Statement

The authors declare that the research was conducted in the absence of any commercial or financial relationships that could be construed as a potential conflict of interest.
